# Extending r/K selection with a maternal risk-management model that classifies animal species into divergent natural selection categories

**DOI:** 10.1038/s41598-019-42562-7

**Published:** 2019-04-16

**Authors:** Deby L. Cassill

**Affiliations:** Department of Biological Sciences, USF St. Petersburg, St, Petersburg, Florida 33701 USA

## Abstract

Reproduction is a defining process of biological systems. Every generation, across all species, breeding females repopulate ecosystems with offspring. *r/K* selection was the first theory to classify animal species by linking the rates with which breeding females repopulated ecosystems, to the stability of ecosystems. Here, I introduce a species classification scheme that extends the reach of *r-K* selection and CSR selection by linking breeder investments in offspring quantity, quality, and diversity to specific natural selection pressures. The species classification scheme is predicated on the assumption that high rates of predation favor breeders that invest more in offspring quantity than quality; and that spatiotemporal scarcity favors breeders that investment more in offspring quality than quantity. I present equations that convert the species classification scheme into a maternal risk-management model. Thereafter, using the equations, I classify eighty-seven animal species into the model’s natural selection categories. Species of reptiles, fish, and marine invertebrates clustered in the *predation selection* category. Species of birds and mammals clustered in the *scarcity selection* category. Several species of apex predators clustered in the *weak selection* category. Several species of social insects and social mammals clustered in the *convergent selection* category. In summary, by acknowledging breeding females as the individuals upon which natural selection acts to repopulate ecosystems with offspring, the proposed maternal risk-management model offers a testable, theoretical framework for the field of ecology.

## Introduction

Just as cells are the basic unit of life, species are the basic unit by which we classify life. The field of taxonomy classifies animal species with shared traits such as body symmetry (asymmetrical, radial, or bilateral) and developmental strategies (holoblastic, meroblastic). In the same vein, the fields of phylogeny and systematics link diverse animal species to common ancestors with shared molecular sequences. Other classification schemes group animal species with shared energy budgets (ectotherm, endotherm), shared diets (herbivore, omnivore, carnivore, insectivore, detritivore), shared reproductive systems (asexual, sexual), or shared mating systems (monogamy, polygamy, promiscuity).

*r/K* selection^1,2^ was the first theory to classify species by linking the rates with which breeding females repopulated ecosystems (*i.e*., potential rates of population growth), to the stability of ecosystems. At one end of the theoretical continuum, stable ecosystems favored the evolution of *K-*selected species, in which breeders invested more in offspring quality than quantity, resulting in slow population growth. At the other end of the continuum, unstable ecosystems favored the evolution of *r*-selected species, in which breeders invested more in offspring quantity than quality, resulting in rapid population growth.

Over decades, field studies investigating breeder investment strategies did not always align with the assumptions or predictions of the *r/K* selection continuum^3–8^. CSR selection^9^ extended *r/K* selection as a unifying theory for ecology. CSR selection classifies species into three ecosystem categories: productive ecosystems, unproductive ecosystems, and lethal ecosystems. Each ecosystem category is associated respectively with competition (C-selection), stress tolerance (S-selection), and regeneration (R-selection).

Here, I introduce a species classification scheme that further extends the reach of *r/K* selection^1,2^ and CSR selection^9^ by linking breeder investment strategies across animal species into divergent natural selection categories. In the following sections, I define the terminology of the species classification scheme, and then illustrate the relationship between breeder investment strategies and natural selection categories with a Punnett square. Next, I disclose the assumptions and equations that transform a conceptual species classification scheme into a predictive, maternal risk-management model. Thereafter, I classify dozens of animal species into the model’s natural selection categories. Lastly, I discuss the ability of the proposed risk-management model to classify animal species that were not addressed by *r/K-*selection^1,2^ or CSR selection^9^.

## Definitions

Breeding females are the focal organisms of the proposed species classification scheme. The term *breeder* refers to fertile females that reproduce sexually or asexually. The term *predation selection* encompasses rates of offspring mortality at multiple levels of biological organization, including predators or scavengers preying on living or dead organisms; parasites preying on organs; microbes preying on cells; and viruses preying on the DNA molecule. The term *predation selection* can also encompass abiotic causes of mortality including floods and fire. The term *scarcity selection* encompasses changes in the spatiotemporal distribution of resources (*i.e*., patchy or seasonal), leading to rates of offspring mortality by starvation, desiccation, or exposure. The term *weak selection* refers to environments with low rates of offspring mortality by predation or scarcity. The term *convergent selection* refers to environments with high rates of offspring mortality by predation and scarcity. The term *offspring diversity* is distilled into a universal trait, fertility. Depending on species, fertility might vary among individuals based on differences in developmental stage, age, size, sex, phenotype, caste, diet, or social status^10–19^.

### Natural selection categories

**Figure 1:**
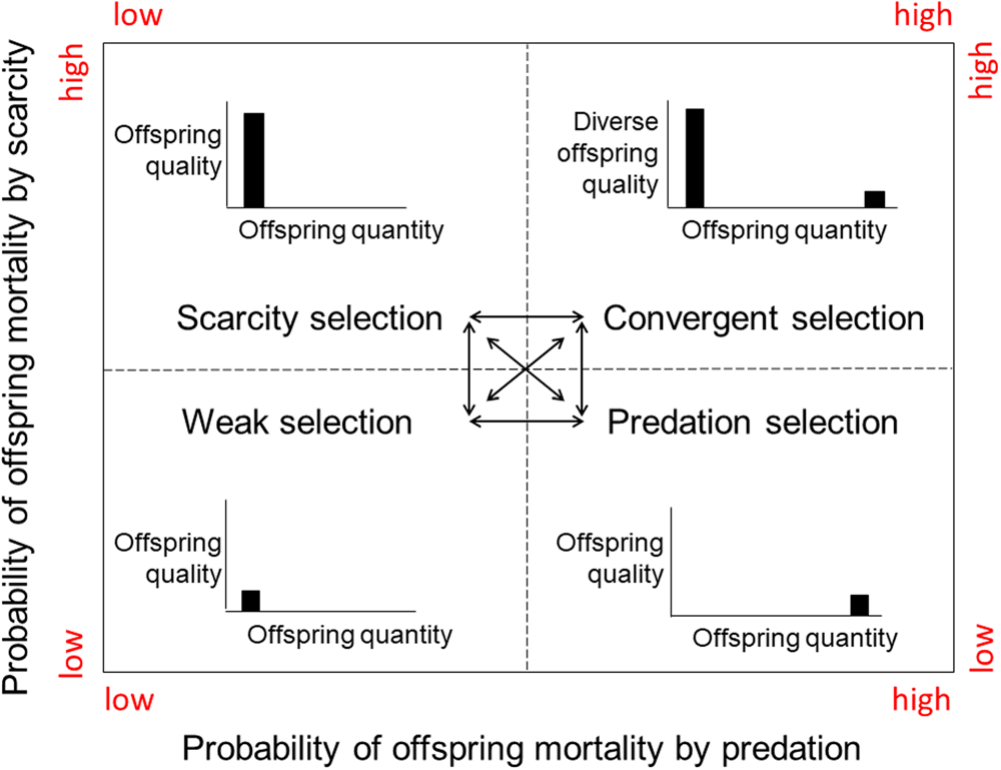
A species classification system for animals. The proposed classification scheme links divergent natural selection processes, predation and scarcity respectively, to the evolution of breeder investments in offspring quantity and offspring quality.

A Punnett square illustrates the conceptual links between breeder investment strategies and divergent natural selection categories (Fig. 1). High rates of offspring mortality in p*redation selection* environments favor breeders that invest more in offspring quantity than quality. Extended maternal care is rare as breeders abandon offspring after spawning. High rates of offspring mortality in *scarcity selection* environments favor breeders that invest more in offspring quality than quantity. To bridge gaps in resources, breeders extend care in temporary, intergenerational family units until offspring are able to disperse or migrate on their own. Alternatives to migration include reduced metabolic processes in the form of cryptobiosis or hibernation. Low rates of offspring mortality in *weak selection* environments favor breeders that invest minimally in offspring quantity and quality. Precocial offspring are abandoned by the breeder after spawn, hatch, or birth. Lastly, high rates of offspring mortality in *convergent selection* environments favor the fusion of breeders and diversified offspring into permanent societies. Division of labor, a hallmark of societies, is an emergent property based on diverse members^16–19^.

### Assumptions

The species classification scheme is predicated on several assumptions about breeder fitness and breeder investment capital. Assumptions are as follows:

#### Breeder fitness (w) is a replacement constant

The term, *replacement*, is defined as the survival of at least two fertile offspring in the next generation: one fertile daughter to replace the breeder; and at least one outbreeding son to replace her mate(s). Within a population of breeders, some will exceed the replacement constant (*w* > 2); some will meet the replacement constant (*w* = 2); and others will fail to meet the replacement constant (*w* < 2). Over generations, as a result of differential replacement success among breeders, populations will expand, contract, diversify, and evolve. Defining breeder fitness as a replacement constant across species (*w* = 2) is a significant deviation from models that have, heretofore, estimated potential breeder fitness based on the number of offspring produced per breeding event or per lifetime^20–22^. Defining fitness as a replacement constant for breeding females eliminates the ambiguity of models that award potential fitness to immature offspring, most of whom do not survive to sexual maturity.

#### Investment capital per breeding event is finite

**Figure 2:**
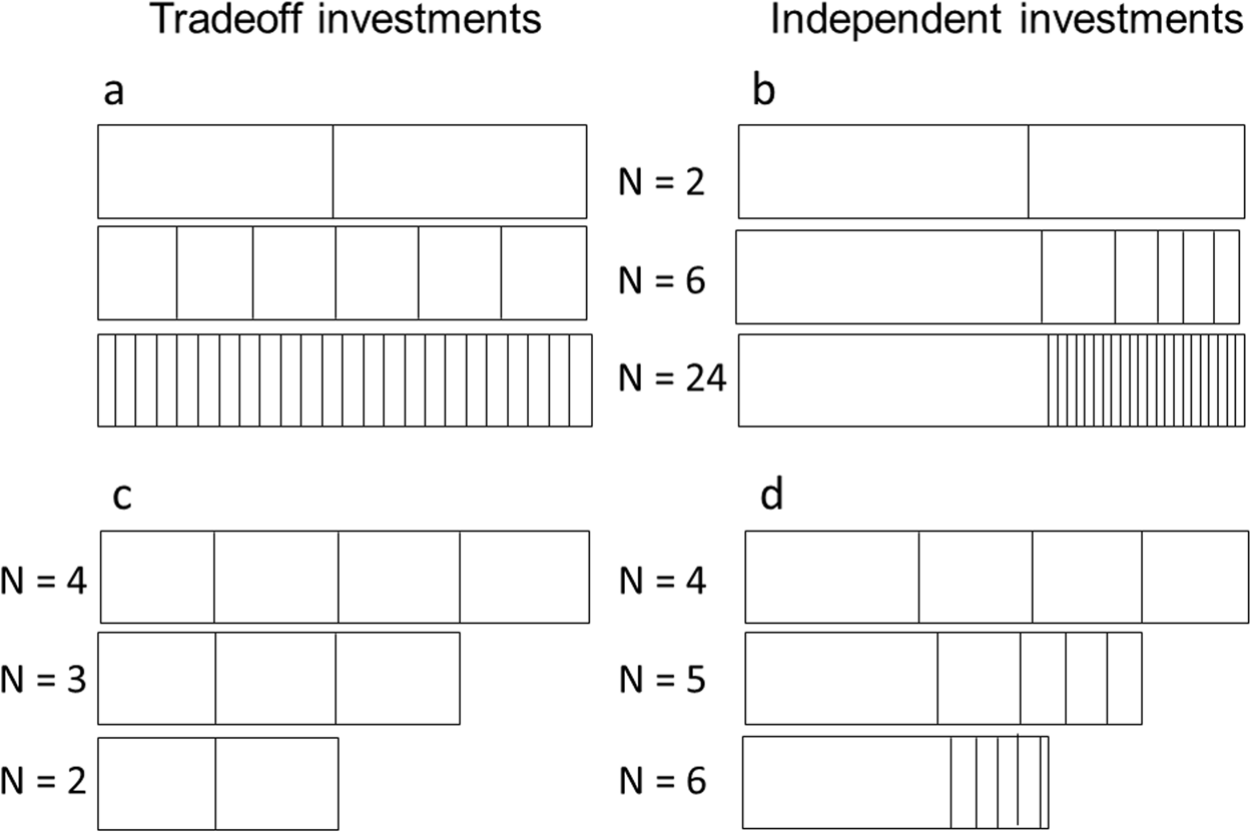
Breeder investment strategies. The outer, horizontal rectangles represent a breeder’s investment capital per breeding event. The inner, vertical rectangles represent offspring size and number per breeding event. (**a**) Tradeoff models assume investments among offspring are equitable. Like dividing a pie into equal servings, the size of a serving predicts the number of servings and vice versa. (**b**) Exploitation models assume breeders invest unequally among offspring. Thus, the size of some offspring can increase without sacrificing offspring number. (**c**) With equal investments among offspring, a reduction in finite resources dictates a reduction in offspring quantity or offspring size. (**d**) With unequal investments among offspring, breeders can increase the quantity and the quality of some offspring, even with a reduction in investment capital.

The assumption that investment capital is finite applies to semelparous species in which females breed once before death, and interoparous species in which females breed multiple times before death. This assumption also applies to capital breeders such as baleen whales and to income breeders such as fire ant queens after their first clutch of eggs hatches into sterile, worker daughters^23–26^. The assumption of finite capital per breeder per breeding event is consistent with *r/K* selection theory^1,2^ (Fig. 2).

#### Investment capital per breeder is independent across multiple breeding events

For iteroparous species, investment capital depends on the availability of resources and the capacity of breeders to accumulate resources over multiple breeding events. A breeder’s investment capital across breeding events varies because luck, more than motivation or ability, determines the accumulation of capital^27^. The assumption of independence across breeding events is a significant deviation from life history’s cost-of-reproduction hypothesis which assumes, *a prior*, a tradeoff between the number of offspring in the first brood versus the last brood^27–29^.

#### Investments in offspring quantity and quality are independent of each other

Depending on the type and intensity of divergent natural selection processes (Fig. 1), breeders might invest equally among offspring^30–32^ (Fig. 2a), or they might diversify investments among offspring^17,18,33^ (Fig. 2b). With independent investment options, breeders are able to increase offspring quantity and the quality of some offspring, even when investment capital is reduced (Fig. 2c versus 2d). The assumption of independent investments in offspring quantity and quality is a significant deviation from tradeoff models such as *r/K* selection theory^1,2^ and bet-hedging theory^30–32^.

In short, by defining breeder fitness as a replacement constant, we are able to ask a question universal to all animal species: *In risky environments, how many, how large, and how diverse must offspring be to ensure the survival of at least two in the next generation; one fertile daughter to replace the breeder and at least one outbreeding son to replace her mate(s)*? In the following section, I connect this three-part question to equations that allow us to classify animal species differently than *r/K* selection^1,2^ or CSR selection^9^. The equations convert the conceptual species classification scheme into a quantitative, maternal risk-management model.

### Equations

The maternal risk-management model is composed of three equations. The first equation addresses the question, “How many offspring should a breeder produce to ensure replacement fitness in *predation selection* environments?” The second equation addresses the question, “How large should offspring be to ensure replacement fitness in *scarce selection* environments?” The third equation addresses the question, “How diverse should offspring be to ensure replacement fitness in *convergent selection* environments?” The equations are as follows:

#### Predation selection

The maternal risk-management model links *predation selection* to breeder investments in offspring quantity as follows:

1 $$P\approx 1-w/N$$

where *P* is the probability of offspring mortality by predation; where *w* is a breeder’s fitness; and where *N* is the number of offspring per clutch, litter, family, or lifetime, depending on the scale of the study.

#### Scarcity selection

The maternal risk-management model links *scarcity selection* to breeder investments in “relative offspring quality” as follows:

2 $$S=m/M$$

where *S* is the rate of offspring mortality by scarcity; and where *S* is also a measure of “relative” offspring quality calculated as a ratio of the mass of offspring (*m*) at dispersal relative to the mass of the breeder (*M*). Relative offspring quality accounts for extended provisioning of offspring by breeders after offspring are expelled from the breeder’s body as eggs, altricial embryos, or precocial young.

#### Convergent selection

The maternal risk-management model links *convergent selection* to diversified breeder investments among offspring as follows:

3 $$C=PS\approx G=[(N+1/N-1)-n/N(N-1){\mu }]({{{\Sigma }}^{N}}_{i=1}{R}_{i}{m}_{i})$$

where *C* is the product of the probabilities of offspring mortality by predation and scarcity (*PS*); where *PS* is a function of *G*; where *G* (the Gini coefficient) is a measure of offspring inequality; where *µ* is the mean size of offspring at dispersal from the mated female, and *R* is the rank of each offspring *i* based on offspring quality *m* at dispersal from the female such that the largest offspring at the time of dispersal receives a rank of 1, and the smallest offspring receives a rank of *N*. The Gini Index is a standardized economic measure of income inequality in which 0.0 represents an equal distribution of resources among offspring and 1.0 represents extreme inequality in the distribution of resources among offspring such that one offspring has 100% of surplus resources and the remaining offspring exist at a subsistence level of survival. The relationship between *convergent selection* pressures and diversified investments by breeders in offspring per clutch, litter, or family unit (Fig. 2) must be confirmed (author, in progress).

### Classifying species

**Figure 3:**
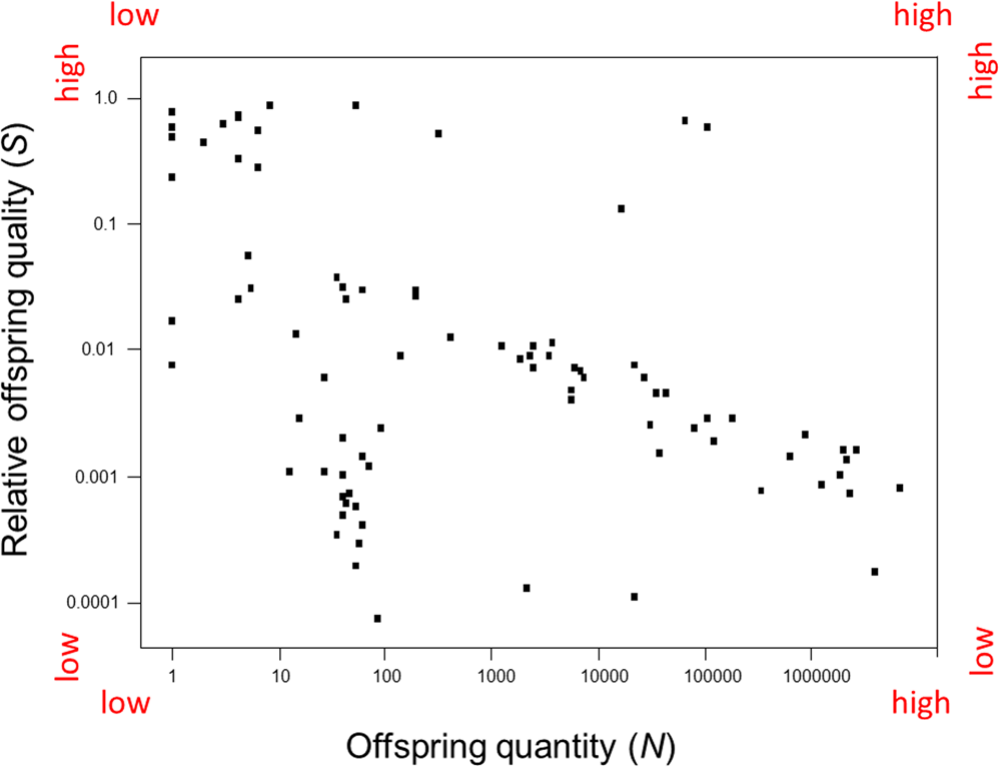
Species classification scheme I. Mean “relative” offspring quality (*S*) and mean offspring quantity (*N*) per breeding event per species were plotted as independent variables (n = 87). Species of animals, ranging from insects to mammals, were not randomly sampled.

**Figure 4:**
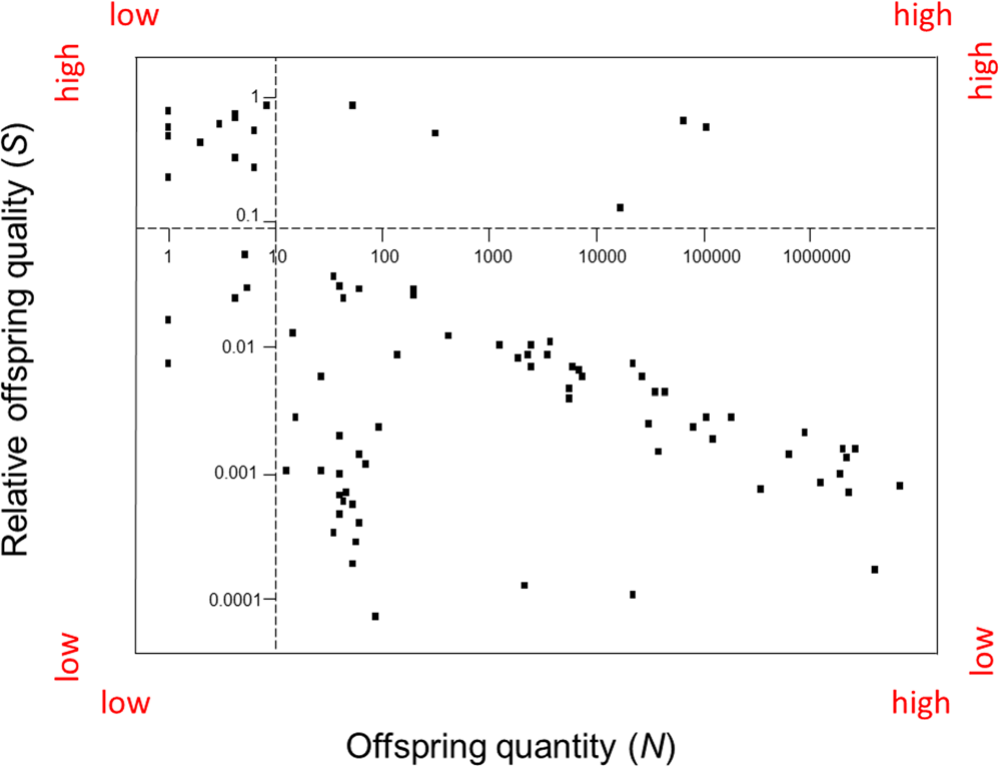
Species classification scheme II. The “y” axis was moved to a set point of 10 on the “x” axis to differentiate large versus small breeder investments in offspring quantity across species. The “x” axis was raised to a set point of 0.1 on the “y” axis to differentiate large versus small breeder investments in relative offspring quality across species. Depending on the scope of the study, set points will differ.

**Figure 5:**
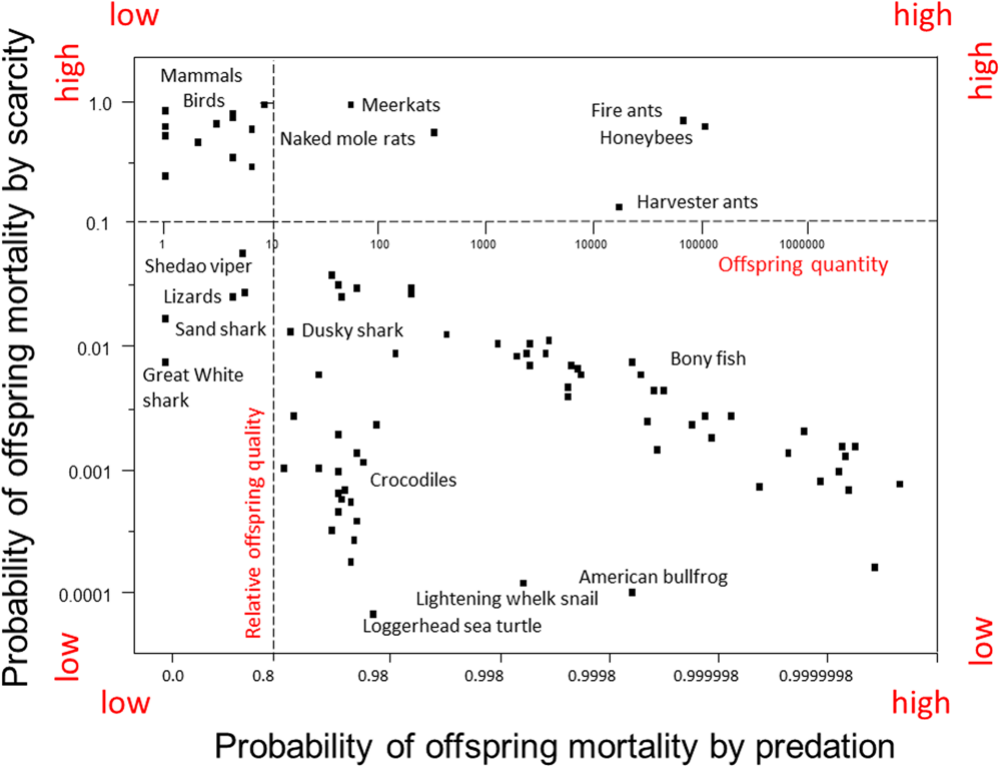
Species classification scheme III. Fish and reptiles were classified as *predation selection* species. Birds and mammals were classifed as *scarcity selection* species. Societies of insects and mammals were classified as *convergent selection* species. Several apex predators were classified as *weak selection* species.

Eighty-seven animal species were classified in three steps. Breeder investments in relative offspring quality were plotted against breeder investments in offspring quantity (Fig. 3). Next, the plot’s “y” and “x” axes were relocated to set points (Fig. 4). Set points will vary depending on the scope of a study. In this study, set points for the *predation selection* category were *S* ≤ 0.1, *N* > 10; set points for the *scarcity selection* category were *S* > 0.1, *N* ≤ 10; set points for the *weak selection* category were *S* ≤ 0.1, *N* ≤ 10; and finally, set points for the *convergent selection* category were *S* > 0.1, *N* > 10. Lastly, using Eqs (1) and (2), the probabilities of offspring mortality by spatiotemporal scarcity and the probabilities of offspring mortality by predation were superimposed over the plot of breeder investments per species (Fig. 5). Species of fish, reptiles, and a marine mollusk clustered in the *predation selection* category. Species of birds and mammals clustered in the *scarcity selection* category. Species of top predators clustered in the *weak selection* category. Species of social vertebrates and social invertebrates clustered in the *convergent selection* category.

The model’s *weak selection* category included species of carnivorous apex-predators and, surprisingly, several species of insectivorous lizards (Fig. 5). To make sense of this juxtaposition, we must account for the scale of each species’ ecological niche. For example, in deep-water marine habitats, Great White sharks are apex predators. On an island off the coast of China, Shedao pit vipers are apex predators^34^. In a thick hedge of bushes or a copse of trees, small lizards are apex predators.

The proposed model’s *convergent selection* category included taxa of social insects and social mammals (Fig. 5). To account for this juxtaposition, I contend that social insects and social mammals converged into the same category, but from different initial categories. Mammals originated in *scarcity selection* environments with ancestral breeders provisioning offspring in temporary family units before *convergent selection* pressures fused multiple family units into permanent societies of diverse members^35–37^. In contrast, insects originated in *predation selection* environments with ancestral breeders investing more in offspring quantity than quality before *convergent selection* pressures fused breeders and their non-dispersing offspring into permanent societies of family members^16–18,38,39^. To my knowledge, this is the first model to hypothesize that convergent natural selection pressures favor the evolution of social species.

## Discussion

*r/K* selection^1,2^ and its successor, CSR selection^9^, were the first to propose a unifying theory for the field of ecology. Here, I compare and contrast the assumptions and predictions of the proposed maternal risk-management model to the assumptions and predictions of *r/K* selection and CSR selection.

**Figure 6:**
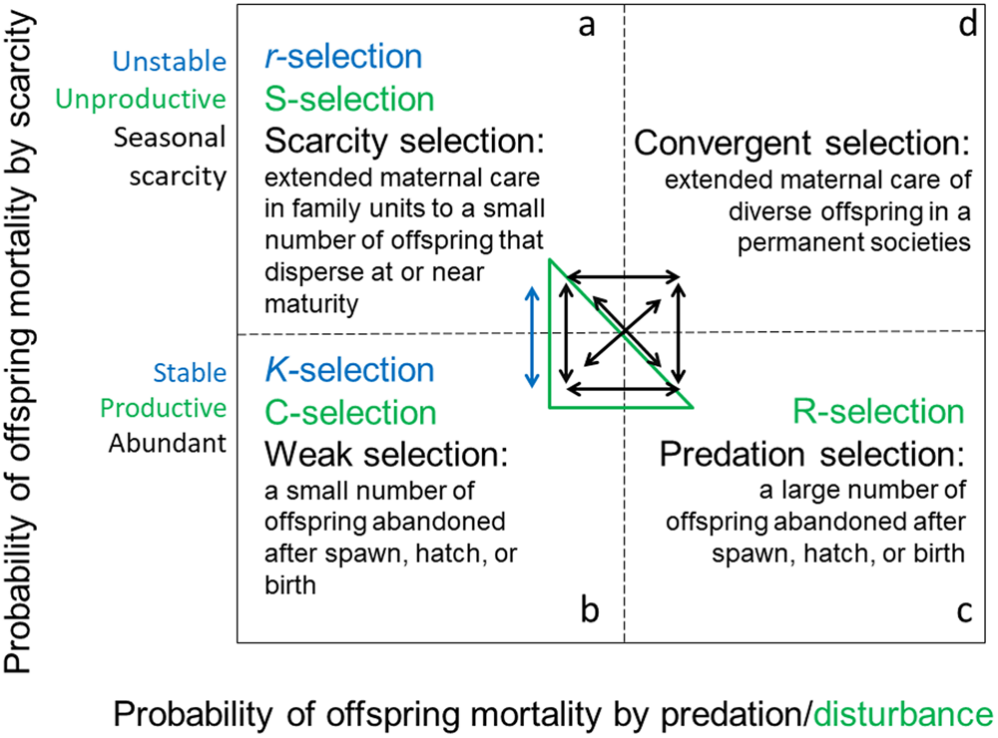
Comparing the maternal risk-management model to *r/K* selection and CSR selection. (**a**) *r-*selection (denoted in blue) and S-selection (denoted in green) are comparable to the proposed model’s *scarcity selection* category (denoted in black). (**b**) *K-*selection (denoted in blue) and C-selection (denoted in green) are comparable to the proposed model’s *weak selection* category (denoted in black). (**c**) R-selection (denoted in green) is comparable to the proposed model’s *predation selection* category (denoted in black). (**d**) Neither *r/K* selection nor CSR selection addresses *convergent selection* (denoted in black). Because offspring quantity and quality are modeled as independent breeder investment strategies, the maternal risk-management model allows for the evolution of offspring quantity and quality in twelve directions rather than the two directions of *r/K* selection or the six directions of CSR selection. In this context, the maternal risk-management model functions as a compass for inferring the natural selection pressures, biotic and abiotic, that shape breeder investments over evolutionary time.

The “y” axis of the maternal risk-management model (Fig. 5), which predicts the probability of offspring mortality on a scarcity continuum from seasonally scarce resources to regularly abundant resources, is equivalent to the *r/K* selection continuum from unstable ecosystems to stable ecosystems (Fig. 6). However, the breeder investment predictions along the *r/K* selection continuum are the inverse of breeder investment predictions of the maternal risk-management model. For example, the maternal risk-management model classifies elephants as scarcity-selected species adapted to seasonal changes in resource availability. In contrast, r/K selection classifies elephants as *K-*selected species adapted to stable ecosystems. Finally, *r/K* selection does not address the *predation selection* or *convergent selection* categories of the maternal risk-management model,

The “y” axis of the maternal risk-management model (Fig. 5), which predicts the probability of offspring mortality on a scarcity continuum from seasonally scarce resources to regularly abundant resources, is equivalent to the S-selection-C-selection continuum from unproductive ecosystems to productive ecosystems (Fig. 6). As with *r/K* selection, the predictions for breeder investments along the S-selection-C-selection continuum are the inverse of those predicted by the proposed model. Elephants were classified as scarcity-selected species adapted to seasonal changes in resource availability rather than C-selected species adapted to productive ecosystems.

The “x” axis of the maternal risk-management model (Fig. 5), which predicts the probability of offspring mortality on a continuum from low predation to high predation, was equivalent to a C-selection-R-selection continuum, ranging from productive ecosystems to lethal ecosystems (Fig. 6). *Predation selection* and R-selection predict that breeders will produce larger numbers of offspring relative to breeders adapted to *weak selection* or C selection environments. Likewise, *predation selection* and R-selection predict that breeders will produce larger numbers of offspring relative to breeders adapted to *scarcity selection* and S-selection. However, CSR selection does not address the *convergent selection* category of the maternal risk-management model.

In summary, by acknowledging that, every generation, breeding females are the individuals upon which natural selection acts to repopulate ecosystems with offspring, the model offers us a conduit through which a unified theory of ecology might be accomplished. Field studies^40,41^, exploring the link between breeder investment strategies and natural selection pressures in other taxa, such as plants, fungi, or protists are needed to determine whether the proposed maternal risk-management model can be extended beyond animal species.

## Methods

Breeder investments in offspring quantity, and relative offspring quality per breeding were compiled for 87 species based on available data from multiple sources. Breeder investments on the Shedao pit viper and fish species were from publications by^34,42^. Breeder investments on Loggerhead sea turtle, *Caretta caretta*, were provided by D. Addison from The Conservancy of Southwest Florida and the Mote Marine Aquarium, Sarasota, Florida, USA. Breeder investments on Crocodilia were from I. Lobaina^43^. Breeder investments on the lizard *Basiliscus vittatus* were provided by J. S. Doody and S. Sullivan. The KwaZulu-Natal Sharks Board, South Africa, provided data on the dusky and spinner sharks, *Carcharhinus obscurus* and *C. brevipinna*. Breeder investments on the red-winged blackbird, *Agelaius phoeniceus*, were provided by S. Forbes. The Florida Fish and Wildlife Research Institute, St. Petersburg, Florida, USA provided egg cases from four lightning whelk snails, *Sinistrofulgur sinistrum*, and a clutch of eggs preserved in formaldehyde from the marine gafftopsail catfish, *Bagre marinus*. The University of Brasilia Herpetology Collection provided the mass of individual legless lizard offspring and the female before returning them to the field. Fertile and sterile members of the fire ant, *Solenopsis invicta*, and the harvester ant, *Pogonomyrmex badius*, were excavated in Pinellas County, Florida, US and weighed by students. Breeder investments for other species where compiled from online sources including zoos, Wikipedia, and state or federal conservation agencies. Mean offspring quantity and quality were calculated when sources differed or when ranges were provided. For species in which breeding females give birth to one offspring per breeding season, offspring quantity was changed to 2.1 to prevent negative numbers when applying replacement fitness (*w* = 2) to calculate the probability of offspring mortality by predation.

## Supplementary information

Breeder investment data per species

## Data Availability

All data generated for this study are available online in Supplement 1, or in an Excel spreadsheet upon request. The acquisition of data on animal breeders and offspring was carried out with relevant guidelines and regulations. Informed consent does not apply.
